# Two Cases of Irritant Poisoning, in One of Which Death Occurred on the Third Day, from Purely Nervous Symptoms, and in the Other on the First Day, with Signs of Violent Inflammation of the Alimentary Canal

**Published:** 1853-03

**Authors:** Jno. C. Dalton


ART. II. — *Two Cases of Irritant Poisoning, in one of which death occurred on the third day, from purely nervous symptoms, and in the other on the first day, with signs of violent inflammation of the alimentary canal.* By JNO. C. DALTON, Jr., M. D.

No. 1. A Case of poisoning with Bichloride of Mercury, inducing delirium tremens. Death on the third day.

L. W., 42 years of age, took poison under the following circumstances: He had been quite intemperate for several years, and had had delirium tremens some time previously. For several days preceding the 12th of August, he had been unable to work on account of debility and a kind of "sick headache." On the forenoon of that day, he had hard words with his wife, who reproached him with lounging about the house, drinking and making himself a burden to her. In consequence of this he procured, at a neighboring apothecary's shop, a little over one ounce of a solution of Bichloride of Mercury, in dilute alcohol, in the proportion of thirty grains to the ounce; giving, as an excuse, that his wife wanted it for "bed-bug poison." The bottle was labeled, in large letters, "ARSENIC.—POISON."

Immediately after obtaining the poison he went home, and at half-past eleven o'clock began pouring it out into a tumbler, in presence of his wife. She endeavored to strike the bottle out of his hand, and in the attempt spilt, as she thinks, about one third of it. He then went down into the cellar and there drank the poison, having (as he said) mixed sugar with it, came up again into the kitchen, placed the bottle and tumbler, both empty, upon the table, and went up stairs to bed. Immediately after swallowing the poison, he experienced, as he said, a burning sensation, which lasted for two hours. He vomited, spontaneously, in about ten minutes. After this, his wife sent for the apothecary, who came immediately and administered an egg, beat up with flour and water, and about fifty grains of powdered ipecac, which operated freely as an emetic. He was first seen by the attending physician, at 12, M. At that time the skin was very cool and the countenance collapsed. The pulse was imperceptible at the wrist. At the carotids it was feeble, and 166 per minute. He took six or seven more eggs, and plenty of flour and water. The vomiting continued. He had, also, frequent dark fluid stools.

He was again seen at 3, P. M., on the same day. He was then lying on his right side, partly prone; but soon changed his position, and often tossed about restlessly. The skin of the face and extremities was cool, damp, a sublivid. The countenance collapsed and anxious. Pulse at the wrist 140

very small, and irregular in force; sometimes strong enough to be counted, sometimes not. The tongue somewhat furred, cool and sticky. Altogether he had much the aspect of a cholera patient, in an early stage of the disease. He did not complain of pain in the abdomen except slightly, just at the time of having a stool; but he complained much of thirst and of "sick headache." The eyes were bright, but he said he was unable to see distinctly. He heard well, however, and answered questions properly. He complained some of occasional cramps in the legs. He was a little inclined to be talkative, reminding one of a patient with delirium tremens. The abdomen was rather sunken, moderately resonant, *not at all tender to pressure anywhere.* The fauces were a little reddened. The patient vomited freely during the visit, nausea being excited by examining the fauces with the blade of a case-knife. He had, also, during the visit, a discharge from the bowels, amounting to about four ounces, and consisting of a thin, watery, dark-reddish fluid, entirely without fæcal odor, and having a quantity of soft, yellowish-brown and whitish flakes floating in it, and also a few minute bloody coagula.

9, P. M., (same day.) Has continued about the same, vomiting frequently after drinking, and having dysenteric discharges, like that during the last visit, about once an hour, without pain. At 6 o'clock he was ordered to take half a grain of opium every hour. He is still restless. Has *no pain*, but complains much of thirst, and insists on drinking, though taking fluid always excites vomiting. His "headache" is much abated, and he feels tolerably easy when lying still, but when he raises his head from the pillow says he has ringing in the ears and some dizziness. Skin about the same, or a little warmer. Pulse stronger, so that it can readily be counted at the wrist, 120. Abdomen as before, absolutely without tenderness on pressure. Patient's manner rather more talkative and flighty than at the last visit, but no positive delirium.

℞. Pulv. Opii. gr. i; and repeat in one hour, unless quiet. Let him have no drink but bits of ice to hold in mouth.

13th, 8, P. M. Patient slept some after two o'clock this morning. In latter part of forenoon the vomiting and purging ceased, but commenced again about 2, P. M. Since then he has vomited often, and been purged twice. He is now lying on the back, and rolling often restlessly from side to side. He has still more the aspect and manner of delirium tremens, though his mind is quite clear. He talks much, and in a quick, hurried manner. He turns his head frequently from one part of the room to another. When questioned, says he does this on account of tendency to vertigo, which is relieved by frequently turning the eyes from one object to another. Has had double vision, occasionally, this P. M. Sight constantly a little dim. Has no pain; but, on raising his head from the pillow, suffers increased dizziness and dimness of vision. Pupils natural, sensible to light. Great coolness of skin on hands and forearms, but less of face. Pulse 108, a little improved in character. Tongue clean, warm, not sticky. Thirst continues, but is less urgent. Patient vomits always after drinking, even a very little. Has also vomited a green, bilious-looking fluid this P. M. Evacuations of the same character as before. No pain with either vomiting or purging, except from straining with the former. Abdomen as before, not all tender. Has passed urine, he says, but once since morning, and then only "a few spoonfuls." He has some soreness of the throat on swallowing, but none along the course of the œsophagus. Has had, to-day, enemata of rice-water and morphine, (gr. $\frac{1}{4}$,) which he refuses to take any longer.

14th, 8, A. M. Delirious. Talks incoherently at times; though, when questioned, answers generally coherently, but in a hurried, irregular manner. Little or no tremor. Has vomited much during the night, and been twice purged. No urine. Has taken about one grain of sulphate of morphine, in divided doses, after which he has shown a disposition to doze for a short time. Coolness of skin about the same. Pulse 100, in character as before. Tongue clean, a little red, dryish. Thirst continues. Abdomen rigid, not tender. *Pupils contracted, insensible to light.* Says he has still double vision at times. No pain. Insists upon having drink as before. Matters vomited of a bilious appearance. Breath very fœtid. Gums swollen and dark-red. No salivation to be observed. Refuses the enemata.

℞. Morphiæ Sulph. gr. j;

Repeat in one hour unless quiet. Let him have a soda-biscuit, and a piece of salt fish.

Patient became somewhat more quiet in the forenoon, but vomited again afterward. Delirium increased. Between 1 and 2, P. M., his wife gave him a piece of soda-biscuit and salt fish, which were not vomited, at least immediately. A little after 4, P. M., he got out of bed and came down the attic stairway on to the first floor. His wife urged him to go back again, telling him "the doctor was coming." He remounted the stairs hastily, and on reaching the top fell down in a fit. During the fit he was convulsed generally and unconscious. He regained consciousness twice, but immediately relapsed into his previous condition. He died in about ten minutes after the second relapse.

*Autopsy* twenty-four hours after death.

Cadaveric rigidity moderately well established. Countenance natural. No remarkable discoloration about the body.

HEAD.

Dura mater natural. Some serous effusion, ℥j. or more, in arachnoid cavity, but the arachnoid membrane itself has a natural appearance. Subarachnoid fluid considerably more abundant than usual.

*Malformation of Falx major.* Falx major of the dura mater is of natural breadth at its commencement at the crista galli, but immediately behind this point is entirely wanting for the anterior quarter of the longitudinal fissure of brain; its place being supplied only by a slightly raised whitish ridge on the lower surface of the dura mater, and a few fibres which run forward and lose themselves in the cellular tissue between the anterior lobes. At the part where the falx is deficient the anterior lobes of the brain are adherent to each other by their plane surfaces, and the arachnoid passes across from right to left, with only a slight depression at the situation of the fissure. No morbid appearances in the neighborhood. The whole of the encephalon is of firm consistency and natural in color, or a little paler than usual. No effusion of fluid into ventricles.

CHEST.

A very small quantity of fluid in the pericardium. Internal surface of the pericardium natural. Right cavities of heart filled with dark-red and fibrinous coagula, and a little fluid blood. The left ventricle well contracted, nearly empty. Left auricle contained a moderate amount of dark fluid blood and coagula. Substance of heart and endocardium natural.

Some old, cellular, pleuritic adhesions of right lung: none of left. Both lungs present a remarkably healthy, well developed appearance, full, rounded, crepitating freely everywhere, and without the slightest induration or atrophy in any part. A moderate post-mortem engorgement of posterior portions.

ABDOMEN.

The peritoneal cavity contains ℥jss. to ℥ij. of thin, watery fluid, slightly tinged with red. Peritoneum itself natural. Intestines of a healthy appearance externally, smooth and shining, of a dull-yellowish color, varied by spots where they are mottled dull-red and purplish. Stomach white externally, with a circular constriction at its central part, by which it is divided into a cardiac and a pyloric portion.

The stomach contained about ℥ij. of a thick, yellowish, gruelly fluid. *The only alteration of the mucous membrane is an increased vascularity;* and this is nothing more than would be produced by the process of digestion. It consists in numerous small, tufted bundles of bright-red vessels, contained in the substance of the mucous membrane. They are most abundant in the great pouch, particularly on its posterior wall, and along the small curvature, where they are numerous for about three-quarters the entire length of the organ. There is a very moderate cadaveric softening and thinning of the mucous membrane in the great pouch; — less than is often seen in cases where no affection of the stomach has been suspected. Toward the pyloric extremity the mucous membrane is entirely natural in color, vascularity, thickness and consistency. The mucous membrane is also stained with bile in some spots in the great pouch and small curvature. There are two small mucous polypi growing from the anterior wall of the organ. Nothing else remarkable. Internal surface of œsophagus natural throughout.

The small intestine contains a little dark-brown and greenish, and rather thick fluid. There is considerable dark-red, vascular congestion in a few spots; and elsewhere the mucous membrane is stained by the contents of the intestine. But there is no ulceration, thickening, nor well-marked softening anywhere. The intestinal glandulæ not at all remarkable. Internal surface of the large intestine also healthy.

The liver was quite pale, but otherwise natural. The gall-bladder contained about ℥ij. of very dark bile. The spleen was very small and quite firm in texture; otherwise natural.

The other abdominal organs were healthy.

This case was somewhat remarkable for the small quantity of the Bichloride (less than thirty grains) which produced death, notwithstanding the appropriate antidotes were immediately administered, and free vomiting took place very soon after the poison was swallowed. The poison seemed to act, also, very much as a mechanical injury would have done, in bringing on delirium tremens in a patient predisposed by his bad habits to this form of disease; and the inflammatory effects of the mercurial salt were, at the same time, very slightly developed or altogether absent, except so far as the intestinal mucous membrane itself was concerned. For although there was frequent vomiting, and abundant dysenteric evacuations, there was no pain of the abdomen, nor tenderness to pressure at any period of the illness. Notwithstanding the late period at which death took place (the third day after swallowing the poison,) the fatal result in this case seems owing to the impression on the nervous system, and not to any local effects of the irritating substance. In fact, the post-mortem appearances would not have given rise to any suspicion of irritant poisoning, if the previous history of the case had been unknown. It should, therefore, be remembered, in judicial investigations, that a patient may die from the effects of a poisonous dose of corrosive sublimate, and yet the intestinal mucous membrane show no inflammatory appearances sufficient to account for death. It is already well understood that this may be the case where a very large dose of the poison has been taken, and death has followed suddenly, at the end of a few hours, in the stage of collapse; and the above case shows that the same thing may happen, under some circumstances, even when death is delayed till the third day. It forms a marked contrast to the following case, in which the whole course and termination of the malady were different.

No. 2. A Case of irritant poisoning. Death on the first day, from collapse. Violent inflammation of the stomach, and some of small intestine, larynx and trachea.

A. G., a married woman, 26 years of age, had been somewhat depressed in spirits owing to the loss of an infant, and other domestic troubles. On the 20th September, she suddenly left home without giving any notice to her friends, and made her way alone to the railroad station of a neighboring city, where she purchased tickets to continue her journey in a direction away from home. A little after 3 o'clock the same afternoon, the station-master observed her to walk quickly through the station-house and enter the ladies' dressing-room. After she had remained there some ten minutes, he sent a female after her to see what she wanted, and learning that she was ill, went in himself, and found her sitting in the water-closet, vomiting freely a kind of "phlegm mixed with white substances." Her lips were very white and swollen. He spoke to her, but she would answer only in monosyllables. She was put into a carriage and conveyed to more comfortable quarters, where she arrived at half-past four. A physician was immediately in attendance.

She was at first able to stand up, and even attempted to get out of the carriage without assistance. Although unable to speak, she evidently understood what was said to her. The skin was cold and damp. Countenance livid. Lips swollen. Pulse imperceptible at the wrists; at the heart, 160. Respiration 40. Eyes suffused. Pupils insensible to light. She was making ineffectual attempts to vomit.

An emetic was prepared for her, but she was unable to swallow it. She rapidly grew more feeble, and was soon unable to stand. At 5 o'clock she vomited about four ounces of a thick, slimy fluid, mixed with reddish flakes; and half an hour afterward had an evacuation from the bowels, of a similar character. Her position was at first bending forward, and indicated distress in the abdomen. She had no marked convulsions, but only once or twice a prolonged *shuddering* motion. The respirations grew gradually slower and more laborious, and the countenance more livid. She died at fifteen minutes past six, P. M.

*Autopsy* — sixteen hours after death.

Cadaveric rigidity very strong. Much purple discoloration of the dependent parts of the body and limbs, and a little of forehead. Excessive lividity of the finger nails. A little frothy fluid, tinged with red, protruding from between the lips. Abdomen natural in appearance externally.

### HEAD.

*Blood in the Arachnoid Cavity.* There was a very small quantity of dark fluid blood in the cavity of the arachnoid, smeared over the posterior part of the right cerebral hemisphere, near the longitudinal sinus. No similar appearance elsewhere. The arachnoid membrane itself, after the blood was wiped off, was natural in appearance. Brain and cerebellum in other respects everywhere healthy.

### CHEST.

There was considerable dryness of the pleura, on both sides of the chest, between the lungs and the pericardium, and between the lungs and the diaphragm. The pericardium contained its ordinary amount of clear fluid.

*Old Pericarditis.* There was a white opaque fringe of old lymph, three-eighths of an inch in length, attached to the anterior surface of the left ventricle, occupying a spot, about a quarter of an inch, or more, in diameter, and having its fringed extremity floating loose in the cavity of the pericardium. Pericardium otherwise healthy.

The right cavities of the heart, and the veins in its immediate neighborhood, contained a very large quantity of dark fluid blood, but no coagula. The left ventricle was firmly contracted.

*Ecchymosis in Heart.* On its internal surface were many spots of dark ecchymosis, situated immediately beneath the endocardium, some of which extended an appreciable distance into the muscular substance of the heart. Heart and endocardium otherwise natural.

*Infiltration and Ecchymosis of Larynx and Trachea.* Some old pleuritic adhesions on both sides of the chest. Much purple œdema about the pharynx and the superior border of the larynx, with spots of ecchymosis immediately beneath the mucous membrane. Some abrasions, also, of the mucous membrane, but no ulcerations. The larynx contained a little semi-transparent and opake mucus. There was much purplish infiltration of the submucous cellular tissue about the aryteno-epiglottidean ligaments, and the upper part of the larynx generally; none of the vocal chords. The posterior surface of the epiglottis was of a clear red color. There was but little infiltration here, but many small circular spots of dark-red ecchymosis, beneath the mucous membrane, particularly toward the right side. The internal surface of the ventricles of the larynx presented the same appearances of infiltration and ecchymosis.

Immediately below the vocal chords the redness and dark purple, submu cous ecchymosis became intense, but there was little or no serous infiltration. This discoloration and ecchymosis continued nearly down to the bifurcation of the trachea on the left side, and a little beyond it on the right, increasing in intensity from above downward. It was everywhere most intense at the anterior part of the trachea, being nearly or quite absent in its posterior, membranous portion. The primary and secondary bronchi were a little redder than usual, but there was no other alteration of their mucous membrane, and no ecchymosis. The bronchi contained a small quantity of frothy, viscid, transparent mucus, slightly tinged with red.

The lungs were everywhere natural ; — only a very moderate bloody engorgement of their posterior portion.

### ABDOMEN.

*Sub-peritoneal Infiltration.* The peritoneum contained two ounces of a clear, thin, claret-colored fluid. The membrane was everywhere natural, except at one spot on the anterior surface of the great pouch of the stomach, where there was a cluster of bright-red streaks of injection or ecchymosis in the peritoneum. There was much reddish infiltration of the cellular and adipose tissue between the layers of the peritoneum along the small curvature of the stomach; and much actual *bloody* infiltration along the large curvature.

The small and large intestines were of a light pinkish color externally; perfectly natural in appearance.

*Inflammation of the Stomach.* The stomach was somewhat contracted and collapsed. It contained about six ounces of a dark-red, bloody-looking fluid, without remarkable odor, which was thickened by holding in suspension a large quantity of light-reddish flakes, like those seen in the discharges of dysentery. There were, also, a few soft masses, like the remains of half-digested food. The parietes of the stomach were generally excessively thickened; so that the anterior wall of the organ, near the pyloric extremity, measured three-eights of an inch in thickness, and near the cardiac extremity, nearly a quarter of an inch. This thickening was entirely owing to infiltration of the submucous cellular tissue with a light-red, serous fluid. This infiltration was nearly uniform throughout the organ. The mucous, muscular and peritoneal coats were not changed in thickness.

The internal surface of the stomach presented an intense purple redness throughout the middle third of the organ, where the mucous membrane was thrown into numerous transverse and longitudinal folds. The redness here existed in the substance of the mucous membrane; and in some spots was so dark and so intense as to constitute a true stain or ecchymosis.

In the great pouch the mucous membrane was darker, but not so red; being of a deep slate color on its free surface, and having a finely *granulated* appearance in consequence of having been strongly corrugated and thrown into minute folds; which had, at the same time, an opake gray color. There was here, also, much dark-purple or black ecchymosis in the substance of the mucous membrane, and beneath it.

In the pyloric portion of the stomach, the mucous membrane was of a dull slaty color, not much reddened, but "mamellonated" and finely granulated, as in the great pouch. (Those portions of the mucous membrane which presented this *grayish* color, had the appearance of being reduced to an eschar, in consequence of uniting chemically with the poisonous substance.)

There was no ulceration, or other morbid appearance beside the above. The œsophagus was of a dull-purple color, internally. There was no alteration of its mucous membrane proper, but considerable purplish-red infiltration of the submucous cellular tissue. Surface of the mucous membrane smeared with a little whitish opake mucus.

*Redness of Intestine.* The upper part of the small intestine contained a fluid similar to that in the stomach; its middle portion a smaller quantity of the same, but lighter colored; the lower part, a much thicker, semi-solid substance, nearly white.

The small intestine had a reddish, slaty color, internally, at the upper part of the duodenum. Below this, the mucous membrane was of a strong brick-red, gradually diminishing in intensity, from above downward, so that there was little or no abnormal redness below the upper third of the intestine.

The intestinal glandulæ were natural.

The large intestine contained three ounces of a fluid, similar to that in the stomach; but there was no marked alteration of its mucous membrane.

Mesenteric glands generally unaltered.

The liver was rather pale and flabby; otherwise natural. The gall-bladder contained a couple of ounces of thinnish, yellowish-brown bile.

The pancreas was natural. The spleen rather large, weighing eight ounces.

Both kidneys were considerably engorged with blood. Other abnormal organs natural.

The blood was everywhere fluid.

Although it was not directly proved, in this case, that the patient took poison, the post-mortem appearances were such as to leave no reasonable doubt of the fact, and at the coroner's inquest it was not thought necessary to order a chemical examination of the contents of the stomach. The woman was known to have corrosive sublimate in her possession, and it was probably this substance which she took for the purpose of self-destruction. The inflammatory appearances in the larynx and trachea are interesting as a somewhat unusual occurrence in these cases. They were probably produced by the contents of the stomach partially finding their way into the air passage during the spasmodic attacks of vomiting.

